# Dietary adenosine 5’-monophosphate supplementation increases food intake and remodels energy expenditure in mice

**DOI:** 10.29219/fnr.v66.7680

**Published:** 2022-06-30

**Authors:** Zifang Wu, Sujuan Rao, Jiaying Li, Ning Ding, Jianzhao Chen, Li Feng, Shuo Ma, Chengjun Hu, Haonan Dai, Lijun Wen, Qingyan Jiang, Jinping Deng, Ming Deng, Chengquan Tan

**Affiliations:** 1 Guangdong Provincial Key Laboratory of Animal Nutrition Control, National Engineering Research Center for Breeding Swine Industry, Institute of Subtropical Animal Nutrition and Feed, College of Animal Science, South China Agricultural University, Guangzhou, Guangdong, China; 2 Guangzhou Customs Technology Center, 510623, China; 3 Tropical Crops Genetic Resources Institute, Chinese Academy of Tropical Agricultural Sciences, Haikou, China; 4 Guangdong Hinabiotech Co., Ltd., Guangzhou, China

**Keywords:** adenosine 5’-monophosphate, food intake, energy expenditure, lipid metabolism

## Abstract

**Background:**

Dietary nucleotides [inclusion adenosine 5’-monophosphate (AMP)] supplementation was shown to promote the feed intake of sows and increase the AMP content in their milk in our previous work, but whether AMP shapes the energy expenditure and lipid metabolism in mammals remains unknown. Here, we aimed to explore the effects and the related mechanism of dietary AMP supplementation on food intake, body composition, energy expenditure, and lipid metabolism in male mice.

**Methods:**

4-week-old C57BL/6 mice (After a 1-wk adaptation) were fed with basal diet and basal diet supplemented with 0.1% AMP, respectively. Animal food intake and body weight were monitored and after 4 weeks all animals were sacrificed to measure the body composition, energy expenditure and lipid metabolism changes.

**Results:**

Compared with the control, the 0.1% AMP fed mice showed higher food intake while lower adipose weight. Intriguingly, dietary AMP supplementation was found to stimulate brown adipose tissue thermogenesis as evidenced by the increase in the uncoupling protein-1 level and the core temperature. Moreover, AMP supplementation was shown to promote white adipose tissue lipolysis as indicated by smaller lipid droplet size in mice. These results demonstrate that dietary AMP supplementation could enhance oxygen consumption and energy expenditure.

**Conclusions:**

This study highlights the physiological importance of AMP supplementation in mediating food intake and energy expenditure and suggests its potential as an adjuvant therapy in preventing energy metabolic disorders (mainly obesity and diabetes).

## Popular scientific summary

- Dietary adenosine 5’-monophosphate supplementation increases the food intake and reduces the body fat content in mice.
- Adenosine 5’-monophosphate stimulates the thermogenesis in brown adipose tissue and the energy expenditure.

Due to the rapid development of social economy and improper lifestyle and diet, the prevalence of obesity has been on the rise over the past few decades (1, 2). Obesity is a major public health problem which could increase the risk of a series of chronic diseases, such as diabetes, cardiovascular disease, and tumours (3–5), suggesting the urgency to adopt measures such as dietary manipulation to prevent obesity and its related diseases.

Obesity is caused by the imbalance of energy metabolism induced by energy intake and consumption, leading to excessive accumulation of adipose, weight gain, and increased body fat rate. According to its functions, the adipose tissue is usually divided into white adipose tissue (WAT), brown adipose tissue (BAT) and beige adipose tissue (6). WAT mainly stores energy in the form of triglycerides, which could be converted into fatty acids and glycerol through lipolysis and released into the blood to provide energy for the body (7, 8), while BAT is mainly involved in thermogenesis [due to the numerous mitochondria and abundant uncoupling protein 1 (UCP1) (9)] to maintain body temperature and energy balance (10). Meanwhile, the beige or brite adipose tissue is derived from WAT browning, with a similar morphology and function to BAT (11, 12). Accumulating evidence indicates that the activation of BAT and WAT browning is a potential treatment strategy against obesity (13–16).

Adenylate functions in mediating energy metabolism, and dietary nucleotide supplementation has been reported to promote the physiological and nutritional functions of animals and improve food intake and lipid metabolism (17–21). In our previous work, dietary nucleotides [inclusion adenosine 5’-monophosphate (AMP)] supplementation were shown to significantly increase the feed intake of sows and the AMP content in their milk. However, whether AMP could influence food intake as well as shape energy expenditure by targeting adipose accumulation remains unknown.

To test this, this study aimed to investigate the effects and the related mechanism of dietary AMP supplementation on the food intake and lipid metabolism in male mice. The results may facilitate the understanding of AMP supplementation in medicating food intake and energy expenditure and provide useful information on its potential use in preventing energy metabolic disorders such as obesity and diabetes.

## Materials and methods

### Animals and diets

All procedures were performed in accordance with the Guidelines for Care and Use of Laboratory Animals of South China Agricultural University (Guangzhou, China) and the experiments were approved by the Animal Ethics Committee of South China Agricultural University (Guangzhou, China).

The 3-week-old C57BL/6J male mice were purchased from Guangdong Medical Laboratory Animal Center (Guangzhou, China). After a 1-wk adaptation, the mice were randomly assigned to two groups (*n* = 16–17), and housed in a light and temperature-controlled facility (12-h light/12-h dark, 22–24°C) with free access to water and food during the experiment. The control group was fed with the basal diet (containing 58% carbohydrate, 18% protein and 4.5% fat) obtained from Guangdong Medical Laboratory Animal Center (Guangzhou, China). The AMP group was fed with the experimental diet prepared by supplementing the basal diet with 0.1% AMP (Nanjing Biotogether Co., Ltd. Nanjing, China). The test lasted 4 weeks, and throughout the experiment, the food intake was measured daily, and body weight was measured twice a week. After treatment for 3 weeks, the meal pattern, locomotor activity (beam breaks) and energy metabolism were monitored for 24 h in a home cage by CLAMS (Promethion Metabolic Screening Systems, Sable systems International, North Las Vegas, NV). During the last week of experiments, the body composition was analysed using a Small Animal Body Composition Analyzer (MesoQMR23-060H, Niumag Corporation, Shanghai, China), the core body temperature (rectal temperature) and BAT temperature by FLIR Quick Report software (FLIR ResearchIR Max 3.4; FLIR), and the core body temperature (rectal temperature) of mice by a rectal probe connected to a digital thermometer (BAT-12, Physitemp, Yellow Spring Instruments). Meanwhile, the blood samples were collected from the upper jaw at 0 and 2 h after meals, and through retro-orbital bleeding at 4 h after meals. Hypothalamus was collected, and the weight was measured for BAT, inguinal white adipose tissues (iWAT), epididymal white adipose tissue (eWAT), tibialis anterior muscle (TA), gastrocnemius muscle (GAS), extensor digitorum longus (EDL), and soleus muscle (SOL). Finally, BAT, iWAT, eWAT, EDL, SOL, TA, GAS, liver, heart, spleen, lung and kidney were collected and stored at −80°C for further use.

### Animal grouping and AMP administration

The 7-week-old C57BL/6J male mice were purchased from Guangdong Medical Laboratory Animal Center (Guangzhou, China), after a 1-wk adaptation, the mice were randomly assigned to five groups, followed by oral gavage administration of 200 μL AMP suspension which contains 3.6 mg AMP [18 mg AMP in 1 mL PBS (Phosphate-buffered saline), to consistent with the daily AMP intake in AMP group], and collecting the blood samples by retro-orbital bleeding at 0, 15, 30, 60 and 120 min after AMP administration. Finally, the contents of AMP and adenosine in the serum were detected by liquid chromatogram-mass spectrometry (LC-MS).

### Glucose tolerance test

For glucose tolerance test (GTT), after fast overnight (20:00 to 08:00) and an intraperitoneal injection of glucose (1 g/kg body weight), the blood glucose concentration of the mice was measured at various time points (0, 30, 60, 90, and 120 min post injection) by using a Sannuo glucometer (San Nuo Inc., Changsha, China).

### Biochemical analysis

Triglyceride (TG), glucose and non-esterified fatty acid (NEFA) in the serum were determined using the commercial kits (A110-1-1, F006-1-1 and A042-2-1; Nanjing Jiancheng Bioengineering Institute, Nanjing, China) according to the manufacturer’s instructions.

### Histologic analysis

The paraffin-embedded BAT and eWAT sections (5–7 μm) were stained with hematoxylin and eosin (H&E), and the adipocyte size (200–300 cells per sample) was quantified using a projecting microscope (Olympus CX41, Japan).

### Real-time quantitative RT-PCR

Total RNA was isolated from tissues and cells using TRIzol reagent (Invitrogen, Carlsbad, USA). cDNA synthesis was performed using 1,000 ng RNA as instructed by the manufacturer for the PrimeScript RT reagent kit (Takara, Dalian, China). Primers used are listed in Supplementary Table 1. Real-time PCR was performed in duplicate on an ABI QuantStudio™ 6 Flex system (Applied Biosystems, Carlsbad, CA). The relative expression of the target genes was calculated by the 2-∆∆Ct method (22).

### Western Blot

The tissues were homogenised in the lysis buffer (RIPA, BioTeke) containing 1 mmol/L protease inhibitor PMSF (Phenylmethylsulfonyl fluoride) (P7626, Sigma) and centrifuged at 12,000 g for 15 min. The supernatant was collected and the protein concentration was measured using BCA Protein Assay Kit (Beyotime Biotechnology, Jiangsu, China). Total protein was separated on SDS–PAGE and blotted onto PVDF (polyvinylidene fluoride) membranes, followed by incubation overnight at 4°C with the following primary antibodies: anti-AgRP (Abcam, USA, 1:1,000 dilution), anti-UCP1 (Abcam, USA, 1:5,000 dilution), anti-POMC (CST, USA, 1:1,000 dilution), and anti-β-actin (CST, USA, 1:1,000 dilution). Finally, the proteins were visualised with the Clarity Max Western ECL Substrate (Bio-Rad, #1705062S), followed by quantification of the density of bands using ImageJ software (National Institutes of Health, Bethesda, MD) and then normalisation to β-actin levels.

### Liquid chromatogram-mass spectrometry

After thawing, samples were fully homogenised by vortexing for 2 min, followed by fully grinding the tissue samples, adding 100 μL of serum or tissue samples in a 1 mL EP tube and removing the protein by adding 500 μL of Methanol/acetonitrile (mass spectrometry grade, 1:1). After vortexing for 2 min, the mixture was centrifuged at 14,500 rpm and 4°C for 15 min, followed by collecting 400 μL of the supernatant and drying under nitrogen at normal temperature. After drying, 200 μL of methanol was added to reconstitute the analyte, followed by vortexing for 2 min and centrifugation at 14,500 rpm and 4°C for 15 min. Finally, the transfer solution was placed in a sample vial (with a liner) and stored at −80°C for further analysis.

The experiments were performed on a Thermo Fisher Scientific UPLC system (Dionex UltiMate 3000) coupled with a mass spectrometer (Q-Exactive Focus). Xcalibur software (version 3.0) was used for instrument control, data acquisition and data analysis.

The chromatographic separation was performed on a C18 Hypersil Gold (100 mm × 2.1 mm, 1.9 μm, Thermo Scientific) column using eluent A (ultrapure water-formic acid 0.1% v/v) and eluent B (ultrapure acetonitrile-formic acid 0.1% v/v) as mobile phase at a flow rate of 0.2 mL/min. The gradient programme was set as follows: 0–7 min, 5–50% B; 7–8 min, 50–75% B; 8–9 min, 75–80% B; 9–11 min, 80–90% B; 11–15 min, 90–95% B; 15–20 min, 95–5% B, with the column temperature of 35°C and the injection volume of 2 μL.

The MS data were acquired using electrospray ionisation (ESI) in the positive ionisation mode, with the ESI parameters of spray voltage 3.5 kV (+3.5 kV in ESI+); sheath gas (N2, >95%), 40 bar; auxiliary gas (N2, >95%), 10 bar; heater temperature, 300°C; capillary temperature, 320°C. Full MS scan ranged from m/z 100 to 1,500 at the resolution of 35,000, and the in-source collision induced dissociation (in-source CID) was set at 0 eV. MS/MS spectra were obtained in data-dependent MS2 (dd-MS2) mode at a resolution of 17,000 with high collision-induced dissociation (HCD) set as stepped mode (10, 30, and 50 eV).

### Statistical analysis

Data are presented as means ± SEM; statistical analyses were performed using SPSS software version 24.0 (SPSS Inc.). The differences between the two groups were analysed by the Student’s *t*-test. The blood kinetic curve of AMP and adenosine were analysed using the one-way analysis of variance (ANOVA) followed by Dunnett’s multiple comparisons test. Differences between groups were considered statistically significant at *P < 0.05, **P < 0.01, ***P < 0.001, and a trend was considered at 0.05 < *P* ≤ 0.10.

## Results

### Dietary AMP supplementation increases the food intake of mice

In this study, we first used the LC-MS method to directly determine the blood kinetic curve of AMP in mice receiving oral gavage of AMP, and found that the content of AMP and adenosine (Which could be converted into AMP (23, 24)) in the serum gradually reached the maximum within 60 min and even could be maintained at 120 min (Supplementary Fig. 1a, b). These findings indicated that AMP could be absorbed into blood and may play a crucial role in modulating the physiological process in the host.

**Fig. 1:**
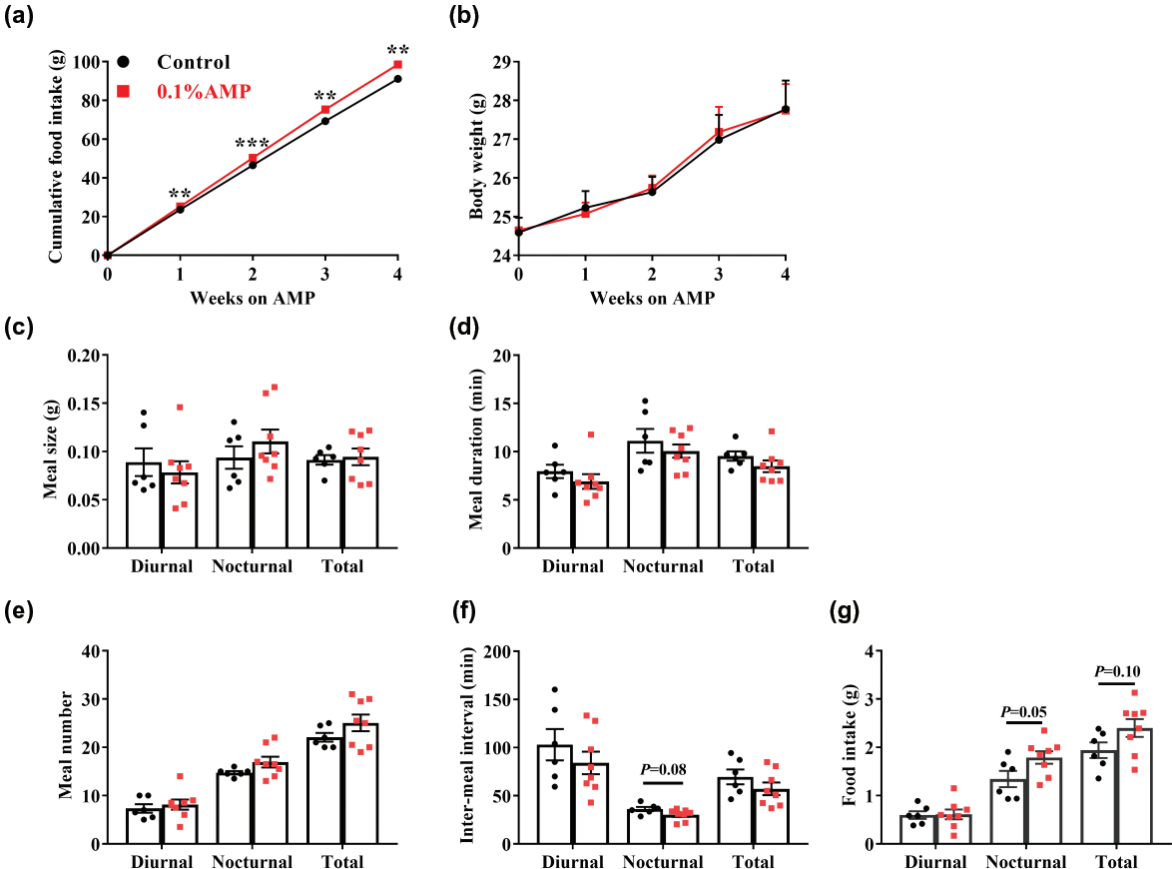
Food intake and meal pattern parameter of mice. (a) Cumulative food intake, (b) Body weight, (*n* = 16–17 in the 1–2 weeks, and *n* = 8–9 in the 3–4 weeks. Half of mice (*n* = 8–9) were assigned to finish the meal pattern detection). (c) Meal size, (d) Meal duration, (e) Meal number, (f) Inter-meal interval, and (g) Food intake (*n* = 6–8). Data are mean ± SEM. Statistical analyses were performed using two-tailed Student’s *t*-test. *P < 0.05, **P < 0.01, ***P < 0.001 versus control. AMP, adenosine 5’-monophosphate.

Subsequently, we investigated the effects of dietary AMP supplementation on the food intake of mice (Fig. 1). The results showed that the cumulative food intake of mice was significantly higher (*P* < 0.01) in the AMP group than in the control group (Fig. 1a), but with no difference (*P* > 0.05) in the body weight (Fig. 1b). Thus, we further explored the meal pattern of mice.

**Fig. 2:**
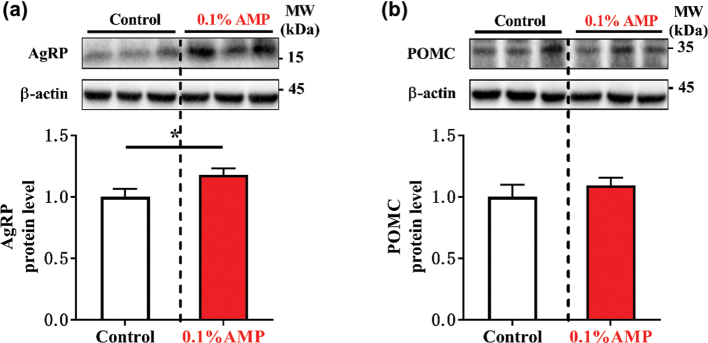
Protein abundance of AgRP (a) and POMC (b) in the hypothalamus of mice. Data are mean ± SEM. Statistical analyses were performed using two-tailed Student’s *t*-test (*n* = 11). *P < 0.05 *versus* control. AgRP, agouti-relative protein; AMP, adenosine 5’-monophosphate; POMC, pro-opiomelanocortin.

Here, we found that despite a similar meal size, meal duration, and meal number (total, diurnal, and nocturnal) (*P* > 0.05) in the two groups (Fig. 1c–e), the AMP group showed a downward trend in nocturnal inter-meal interval (*P* = 0.08) (Fig. 1f) and an upward trend in nocturnal food intake (*P* = 0.05) (Fig. 1g). Moreover, we also observed that dietary AMP supplementation could increase the level of the orexin-stimulating protein AgRP in hypothalamus (Fig. 2a), but with little effect on the level of the orexin-inhibiting protein POMC (Pro-opiomelanocortin) (Fig. 2b). The aforementioned findings suggest that dietary AMP supplementation could promote the food intake of mice mainly by enhancing appetite.

### Dietary AMP supplementation affects the concentration of nutritional substrates in the serum of mice

**Fig. 3:**
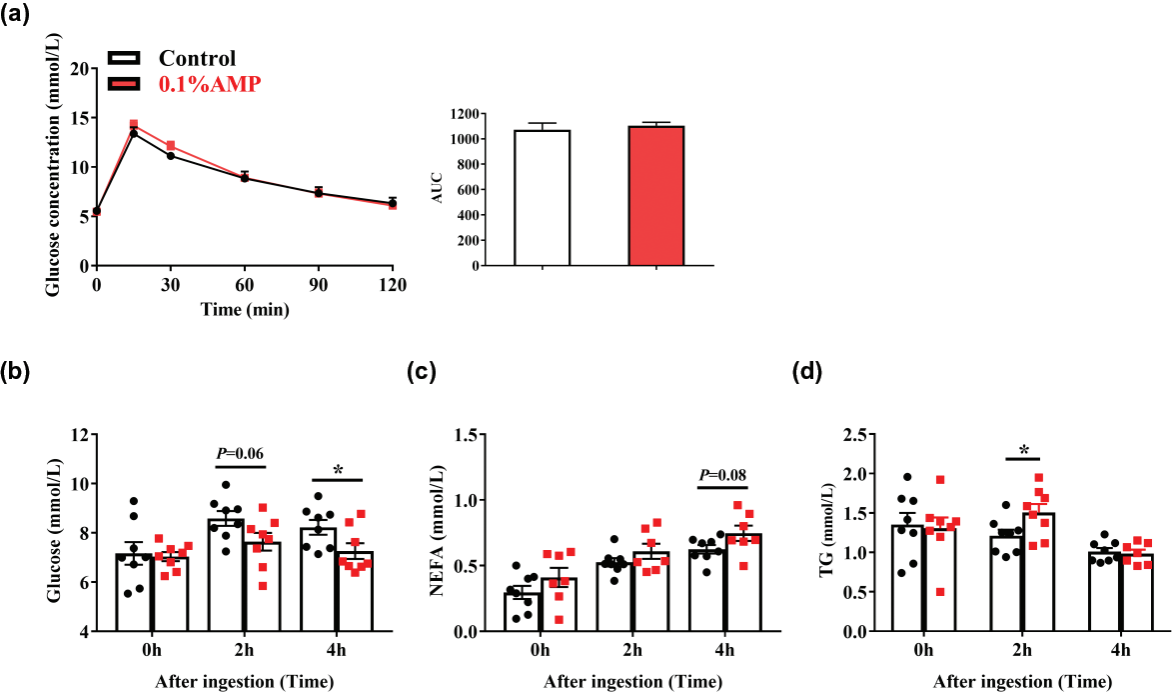
Serum nutritional substrates of mice. (a) Glucose response curve during the glucose tolerance test and the AUC for glucose level. (b) Glucose, (c) NEFA, and (d) TG content in serum, (*n* = 7–8). Data are mean ± SEM. and statistical analyses were performed using two-tailed Student’s *t*-test. *P < 0.05 versus control. AMP, adenosine 5’-monophosphate; AUC, area under the curve; NEFA, non-esterified fatty acid; TG, Triglyceride.

After considering the fact that AMP treatment could highly increase the food intake of mice, we next investigated whether AMP could alter the nutritional substrates in the serum of mice. Interestingly, AMP treatment showed little effect on the results of the area-under-the-curve (AUC) analysis of GTT in the two groups (Fig. 3a). However, compared with the control, the AMP group showed a decrease in the serum concentrations of glucose at both 2 h (*P* = 0.06) and 4 h (*P* < 0.05) (Fig. 3b), in contrast to an increase in the level of NEFA (*P* = 0.08) and TG (*P* < 0.05) (Fig. 3c–d). These observations indicate that dietary AMP supplementation may increase the concentration of NEFA and TG and decrease the concentration of glucose in the postprandial serum of mice.

### Dietary AMP supplementation shapes the body composition of mice

**Fig. 4:**
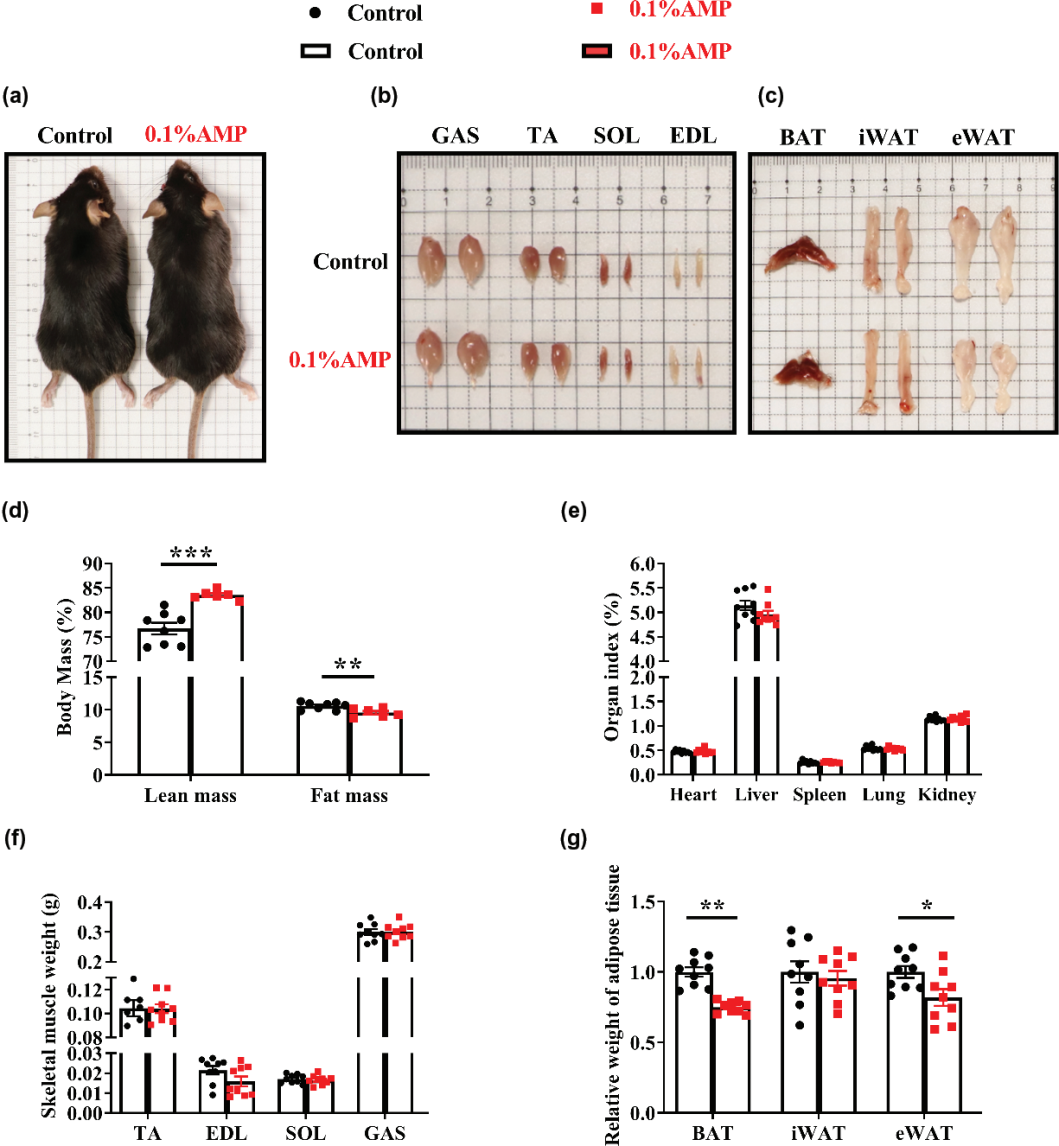
Body composition of mice. The representative image of mice (a), the skeletal muscles (b), the adipose tissue (c), body mass (d) in mice, the organ index (e), the skeletal muscle weight (f), and the relative weight of adipose tissue (g). Data are mean ± SEM. Statistical analyses were performed using two-tailed Student’s *t*-test, (*n* = 6–9). *P < 0.05, **P < 0.01, ***P < 0.001 *versus* control. AMP, adenosine 5’-monophosphate; BAT, brown adipose tissue; EDL, extensor digitorum longus; eWAT, epididymal white adipose tissue; GAS, gastrocnemius; iWAT, inguinal white fat tissue; SOL, soleus; TA, tibialis anterior.

Given that dietary AMP supplementation increased food intake without affecting body weight, we subsequently investigated the effects of dietary AMP supplementation on the body composition of mice (Fig. 4a–c). In terms of body mass, we found that AMP highly increased the percentage of lean mass while reducing the percentage of fat mass (Fig. 4d). However, the two groups showed no significant difference in organ index (including heart, liver, spleen, lung, and kidney) (Fig. 4e) and skeletal muscle weight (including TA, EDL, SOL, and GAS) (Fig. 4f). It is worth noting that AMP supplementation could significantly lower the relative weight of adipose tissue (chiefly BAT and eWAT) (Fig. 4g). These results indicate that dietary AMP supplementation could induce body composition remodeling by decreasing fat mass and increasing lean mass in mice.

### Dietary AMP supplementation promotes the energy expenditure of mice

**Fig. 5:**
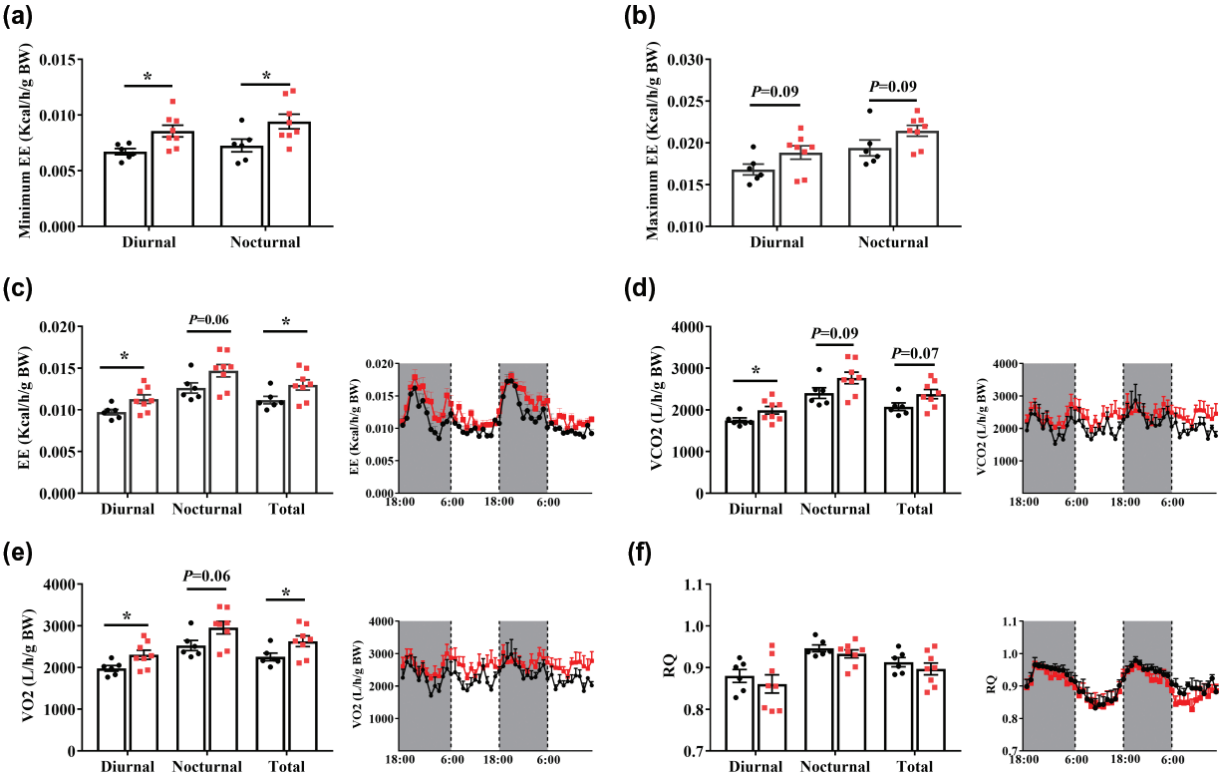
Energy expenditure of mice. Minimum energy expenditure (a), maximum energy expenditure (b), average energy expenditure (c) of mice, (d–f) carbon dioxide production (d), oxygen consumption (e), and respiratory quotient (f) of mice (*n* = 6–8). Data are mean ± SEM. Statistical analyses were performed using two-tailed Student’s *t*-test. *P < 0.05 *versus* control. AMP, adenosine 5’-monophosphate; EE, energy expenditure; VCO_2_, carbon dioxide production; VO_2_, oxygen consumption; RQ, respiratory quotient.

As the decreased body fat rate may result from enhanced energy metabolism and thermogenesis, we explored whether AMP supplementation promoted energy metabolism and thermogenesis. In Fig. 5, the AMP group was shown to have significantly (*P* < 0.05) higher energy expenditure than the control (Fig. 5a–c). Meanwhile, the AMP group showed an increase (*P* < 0.05) in oxygen consumption and carbon dioxide production throughout the day (Fig. 5d, e). Collectively, dietary AMP supplementation could promote energy and lipid metabolism in mice.

### Dietary AMP supplementation enhances the thermogenesis and lipid metabolism of BAT in mice

**Fig. 6:**
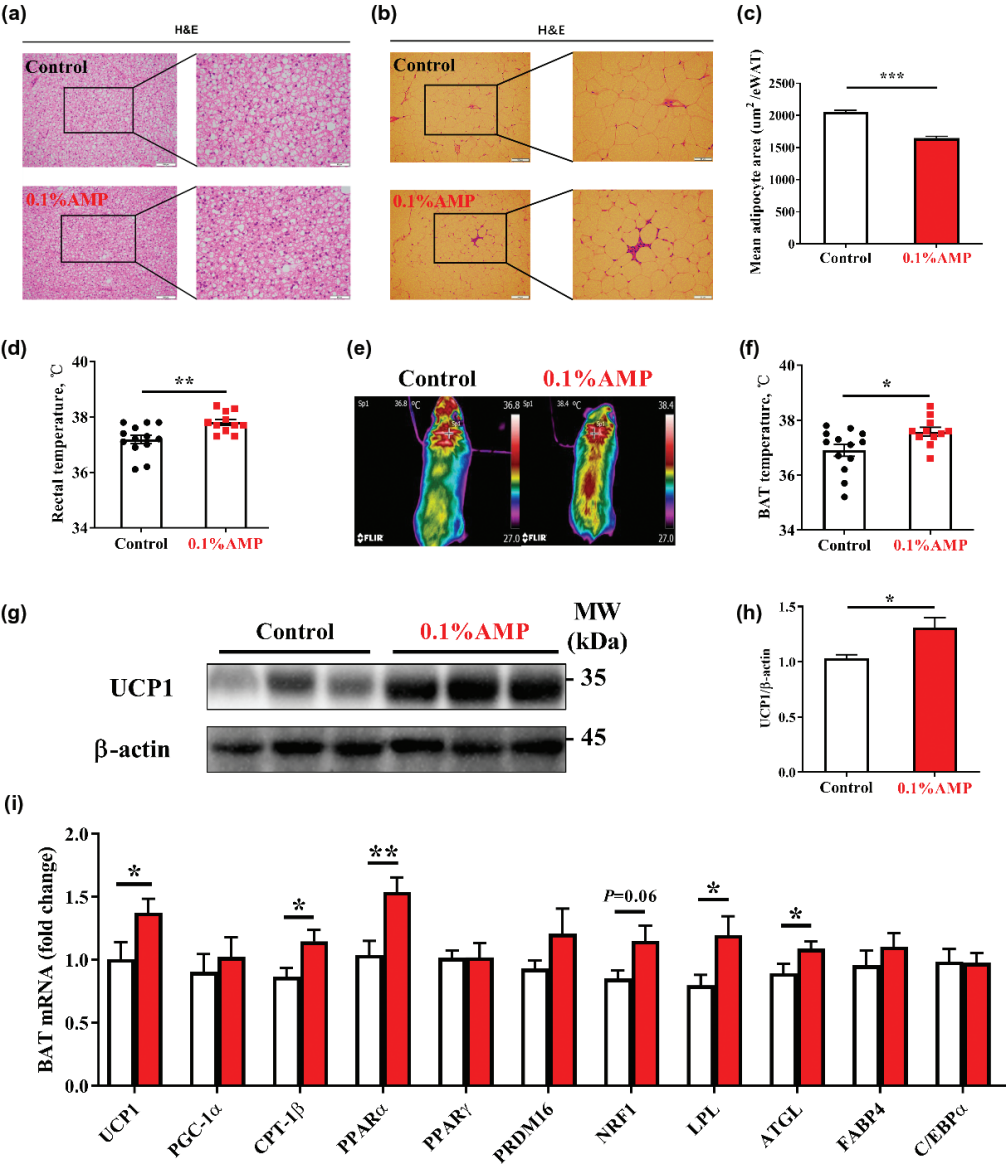
Thermogenesis of mice. (a, b) The representative image of BAT (a) and eWAT (b), (c) The mean adipocyte area of eWAT. Scale bar: 100 μm (a, b) and 50 μm (a, b) in reprehensive picture, (d) The core body temperature (rectal temperature) of mice, (e, f) the dorsal view (e) of mice by IR thermography, and the BAT temperature (f) of mice, (g, h) the relative protein expression of UCP1 in BAT, (*n* = 11–13), and (i) Relative mRNA expression of lipid metabolism genes in BAT, (*n* = 9–10). Data are mean ± SEM. Statistical analyses were performed using two-tailed Student’s *t*-test. *P < 0.05, **P < 0.01, ***P < 0.001 *versus* control. AMP, adenosine 5’-monophosphate; BAT, brown adipose tissue; eWAT, epididymal white adipose tissue; H&E, hematoxylin and eosin staining.

More importantly, AMP supplementation was shown to reduce the size of lipid droplets in BAT and the mean adipocyte area of eWAT (Fig. 6a–c). Additionally, the AMP group was also significantly higher than the control group in rectal body temperature and BAT temperature (Fig. 6d–f). The increased adipose consumption is usually related to the enhanced BAT thermogenesis. Thus, we investigated the expression of key genes for lipid metabolism in BAT. Notably, the AMP group was significantly higher (*P* < 0.05) than the control in the relative protein level of UCP1 in BAT (Fig. 6g, h) and the relative mRNA level of lipid metabolism genes (*UCP1, CPT-1β, PPARα, LPL, and ATGL*) (Fig. 6i). These results suggest that AMP supplementation could increase the activation of BAT in mice.

## Discussion

Despite extensive research about the effects of AMP supplementation on the food intake in aquaculture (18, 25, 26), there are few reports regarding the use of AMP as an additive in mammal diets (27–30). Furthermore, to our knowledge, the effect of AMP diet on mammal food intake and energy metabolism has not been reported yet. In our previous study, we found that supplementation of 1 g/kg nucleotides in the diet can significantly increase the feed intake in sows (31, 32). Later, we analysed the diet composition under the supplementation of nucleotides, and interestingly, we found a significant increase of AMP content in the diet and the milk of the nucleotide group versus the control group (31, 32). In the current study, dietary AMP supplementation was shown to increase the food intake by promoting the expression of AgRP and shortening the feeding interval, which is consistent with a previous study in aquatic (18), which reported that dietary 0.1% AMP supplementation contributed to the food intake of red sea bream (18). This can be attributed to the reason that the adenosine in food AMP can be absorbed into the body and reduce the concentration of glucose after meal (33, 34). Studies have also shown that high levels of glucose after a meal reduce the AMP/ATP ratio in rats (35), or also reduce the level of AgRP in isolated hypothalamic cultures (36). This view was well supported by the protein expression of AgRP in the present study. In summary, dietary AMP supplementation has a promoting effect on feeding, which we speculate may be related to the AMP ingestion induced decrease in blood glucose in the body (34), leading to an increase in the AMP/ATP ratio (35), promoting the protein expression of AgRP by activation of protein kinase activated by adenosine monophosphate (AMPK) (36), and finally an increase in the food intake in mice.

AMP is known as a purine nucleotide and participant in ATP metabolism. Studies have proved that AMP can be absorbed into the circulatory system and participate in metabolism (33, 37). Here, we also found that AMP can be absorbed into the blood from the serum kinetic curve, which is consistent with the previous studies (33). A possible explanation for this phenomenon is that AMP is absorbed into the blood and enriched in the adipose tissue to play a subsequent regulatory role. The mice in the 0.1% AMP group were noted to display higher food intake but constant body weight gain compared to the control group. Energy expenditure was reported to be closely related to the spontaneous physical activity and basal metabolic of animals (10). To explore the direct effect of dietary AMP supplementation on the body weight gain, we tested the body composition and energy expenditure of the mice. Surprisingly, we found that AMP treatment decreased fat mass including BAT and eWAT, and increased the lean mass. Meanwhile, AMP treatment also significantly increased the energy metabolism of mice, especially the basic energy expenditure.

Both WAT and BAT make up the adipose organ and regulated the energy balance of the body, with WAT as the primary site of energy storage, and BAT to facilitate adaptive thermogenesis and increase metabolic rate (38, 39). We suspect that the increased energy expenditure caused by AMP is related to the thermogenesis of BAT and lipolysis of WAT. Thus we explored the adipocytes and thermogenesis in mice, and found that AMP could reduce the size of lipid droplets in BAT and the mean adipocyte area of eWAT, increase the temperature of BAT and rectum, and promote thermogenesis and lipolysis of BAT. All these observations imply that AMP reduces adipose by increasing body energy consumption and adipose heat production while promoting food intake. To prove the relation between adipose reduction and BAT activity, we tested the expression of thermogenesis and lipolysis genes in BAT, and found that adipose reduction co-occurred with the up-regulation of thermogenesis and lipolysis genes in BAT. Previous studies have also shown that AMP analogs and BAT activation reduce the volume of adipocytes (40, 41), and the expression of UCP1, PPARα, CPT-1β in BAT is closely related to BAT activation (42–46). On the other hand, the expression of ATGL and LPL also participated in the lipolysis and thermogenesis regulated by BAT (47–50). This conclusion was well supported by our results.

In conclusion, dietary AMP supplementation increases the food intake and reduces the body fat content of mice without affecting body weight and organs index. In addition, AMP reduces adipose content and increases lean meat content by promoting BAT activation and thermogenesis without affecting body weight. These results contribute to a better understanding of the role of dietary AMP supplementation in promoting food intake and lipid metabolism, and suggest that AMP supplementation may be a potential strategy to treat obesity by lowering adipose without affecting body weight and health, which provides inspiration for obesity treatment.

## Supplementary Material

Dietary adenosine 5’-monophosphate supplementation increases food intake and remodels energy expenditure in mice

## References

[1] Malik VS, Willet WC, Hu FB. Nearly a decade on – trends, risk factors and policy implications in global obesity. Nat Rev Endocrinol 2020; 16(11): 615–6. doi: 10.1038/s41574-020-00411-y. PubMed PMID: WOS:000565173100001. 32873971 PMC7461756

[2] De Lorenzo A, Romano L, Di Renzo L, Di Lorenzo N, Cenname G, Gualtieri P. Obesity: a preventable, treatable, but relapsing disease. Nutrition 2020; 71: 110615. doi: 10.1016/j.nut.2019.110615. PubMed PMID: 31864969. 31864969

[3] Akhlaghi M. Dietary approaches to stop hypertension (DASH): potential mechanisms of action against risk factors of the metabolic syndrome. Nutr Res Rev 2020; 33(1): 1–18. doi: 10.1017/s0954422419000155. PubMed PMID: WOS:000531843200001. 31358075

[4] Jastreboff AM, Kotz CM, Kahan S, Kelly AS, Heymsfield SB. Obesity as a disease: the obesity society 2018 position statement. Obesity 2019; 27(1): 7–9. doi: 10.1002/oby.22378. PubMed PMID: WOS:000453750700001. 30569641

[5] Malik VS, Willett WC, Hu FB. Global obesity: trends, risk factors and policy implications. Nat Rev Endocrinol 2013; 9(1): 13–27. doi: 10.1038/nrendo.2012.199. PubMed PMID: WOS:000312387900006. 23165161

[6] Sun W, Dong H, Balaz M, Slyper M, Drokhlyansky E, Colleluori G, et al. snRNA-seq reveals a subpopulation of adipocytes that regulates thermogenesis. Nature 2020; 587(7832): 98. doi: 10.1038/s41586-020-2856-x. PubMed PMID: WOS:000582810000003. 33116305

[7] Coelho M, Oliveira T, Fernandes R. Biochemistry of adipose tissue: an endocrine organ. Archiv Med Sci 2013; 9(2): 191–200. doi: 10.5114/aoms.2013.33181. PubMed PMID: WOS:000317994900001. PMC3648822 23671428

[8] Medina-Gomez G. Mitochondria and endocrine function of adipose tissue. Best Pract Res Clin Endocrinol Metab 2012; 26(6): 791–804. doi: 10.1016/j.beem.2012.06.002. PubMed PMID: WOS:000312466500008. 23168280

[9] Inagaki T, Sakai J, Kajimura S. Transcriptional and epigenetic control of brown and beige adipose cell fate and function. Nat Rev Mol Cell Biol 2016; 17(8): 480–95. doi: 10.1038/nrm.2016.62. PubMed PMID: WOS:000380745800008. 27251423 PMC4956538

[10] Marlatt KL, Ravussin E. Brown adipose tissue: an update on recent findings. Curr Obes Rep 2017; 6(4): 389–96. doi: 10.1007/s13679-017-0283-6. PubMed PMID: WOS:000417072000005. 29101739 PMC5777285

[11] Ikeda K, Maretich P, Kajimura S. The common and distinct features of brown and beige adipocytes. Trends Endocrinol Metab 2018; 29(3): 191–200. doi: 10.1016/j.tem.2018.01.001. PubMed PMID: WOS:000425589200007. 29366777 PMC5826798

[12] Wu J, Bostrom P, Sparks LM, Ye L, Choi JH, Giang A-H, et al. Beige adipocytes are a distinct type of thermogenic fat cell in mouse and human. Cell 2012; 150(2): 366–76. doi: 10.1016/j.cell.2012.05.016. PubMed PMID: WOS:000306595700015. 22796012 PMC3402601

[13] Alipoor E, Hosseinzadeh-Attar MJ, Rezaei M, Jazayeri S, Chapman M. White adipose tissue browning in critical illness: a review of the evidence, mechanisms and future perspectives. Obes Rev 2020; 21(12): e13085. doi: 10.1111/obr.13085. PubMed PMID: WOS:000544280200001. 32608573

[14] Fernandez-Verdejo R, Marlatt KL, Ravussin E, Galgani JE. Contribution of brown adipose tissue to human energy metabolism. Mol Aspects Med 2019; 68: 82–9. doi: 10.1016/j.mam.2019.07.003. PubMed PMID: WOS:000482103700007. 31306668 PMC7112661

[15] Abdullahi A, Jeschke MG. Taming the flames: targeting white adipose tissue browning in hypermetabolic conditions. Endocr Rev 2017; 38(6): 538–49. doi: 10.1210/er.2017-00163. PubMed PMID: WOS:000419059200003. 28938469 PMC5716828

[16] Carey AL, Kingwell BA. Brown adipose tissue in humans: therapeutic potential to combat obesity. Pharmacol Ther 2013; 140(1): 26–33. doi: 10.1016/j.pharmthera.2013.05.009. PubMed PMID: WOS:000325123500003. 23718981

[17] Jang KB, Kim SW. Supplemental effects of dietary nucleotides on intestinal health and growth performance of newly weaned pigs. J Anim Sci 2019; 97(12): 4875–82. doi: 10.1093/jas/skz334. PubMed PMID: WOS:000507889900018. 31665463 PMC6915224

[18] Hossain MS, Koshio S, Ishikawa M, Yokoyama S, Sony NM. Dietary effects of adenosine monophosphate to enhance growth, digestibility, innate immune responses and stress resistance of juvenile red sea bream, Pagrus major. Fish Shellfish Immunol 2016; 56: 523–33. doi: 10.1016/j.fsi.2016.08.009. PubMed PMID: WOS:000383292200058. 27514786

[19] Martinez-Puig D, Manzanilla EG, Morales J, Borda E, Perez JF, Pineiro C, et al. Dietary nucleotide supplementation reduces occurrence of diarrhoea in early weaned pigs. Livest Sci 2007; 108(1–3): 276–9. doi: 10.1016/j.livsci.2007.01.099. PubMed PMID: WOS:000247123100068.

[20] Carver JD. Dietary nucleotides: effects on the immune and gastrointestinal systems. Acta Paediatr 1999; 88: 83–8. doi: 10.1080/080352599750029790. PubMed PMID: WOS:000083296700015. 10569229

[21] Lopez-Navarro AT, Ortega MA, Peragon J, Bueno JD, Gil A, Sanchez-Pozo A. Deprivation of dietary nucleotides decreases protein synthesis in the liver and small intestine in rats. Gastroenterology 1996; 110(6): 1760–9. doi: 10.1053/gast.1996.v110.pm8964401. PubMed PMID: BIOSIS:PREV199699059291. 8964401

[22] Duan Y, Li F, Tan B, Lin B, Kong X, Li Y, et al. Myokine interleukin-15 expression profile is different in suckling and weaning piglets. Anim Nutr 2015; 1(1): 30–5. doi: 10.1016/j.aninu.2015.02.005. PubMed PMID: MEDLINE: 29766983. 29766983 PMC5884465

[23] Alonso-Andres P, Albasanz JL, Ferrer I, Martin M. Purine-related metabolites and their converting enzymes are altered in frontal, parietal and temporal cortex at early stages of Alzheimer’s disease pathology. Brain Pathol 2018; 28(6): 933–46. doi: 10.1111/bpa.12592. PubMed PMID: WOS:000457460300012. 29363833 PMC8028663

[24] Fredholm BB. Adenosine-a physiological or pathophysiological agent? J Mol Med 2014; 92(3): 201–6. doi: 10.1007/s00109-013-1101-6. PubMed PMID: WOS:000334268800002. 24362516

[25] Ming D, Ninomiya Y, Margolskee RF. Blocking taste receptor activation of gustducin inhibits gustatory responses to bitter compounds. Proc Natl Acad Sci U S A 1999; 96(17): 9903–8. doi: 10.1073/pnas.96.17.9903. PubMed PMID: BIOSIS:PREV199900415233. 10449792 PMC22308

[26] Kiyohara S, Hidaka I, Tamura T. The anterior cranial gustatory pathway in fish. Experientia 1975; 31(9): 1051–3. doi: 10.1007/bf02326954. PubMed PMID: MEDLINE: 1175743. 1175743

[27] Liu YL, Zhang YM, Yin J, Ruan Z, Wu X, Yin YL. Uridine dynamic administration affects circadian variations in lipid metabolisms in the liver of high-fat-diet-fed mice. Chronobiol Int 2019; 36(9): 1258–67. doi: 10.1080/07420528.2019.1637347. PubMed PMID: WOS:000476378100001. 31296061

[28] Tie HM, Wu P, Jiang WD, Liu Y, Kuang SY, Zeng YY, et al. Dietary nucleotides supplementation affect the physicochemical properties, amino acid and fatty acid constituents, apoptosis and antioxidant mechanisms in grass carp (Ctenopharyngodon idellus) muscle. Aquaculture 2019; 502: 312–25. doi: 10.1016/j.aquaculture.2018.12.045. PubMed PMID: WOS:000455344800039.

[29] Hoang Do H. Overview of the application of nucleotide in aquaculture. J Coast Life Med 2016; 4(10): 816–23. doi: 10.12980/jclm.4.2016J6-165. PubMed PMID: BIOSIS:PREV201600799008.

[30] Masic U, Yeomans MR. Umami flavor enhances appetite but also increases satiety. Am J Clin Nutr 2014; 100(2): 532–8. doi: 10.3945/ajcn.113.080929. PubMed PMID: WOS:000339599100006. 24944058

[31] Tan C, Ji Y, Zhao X, Xin Z, Li J, Huang S, et al. Effects of dietary supplementation of nucleotides from late gestation to lactation on the performance and oxidative stress status of sows and their offspring. Anim Nutr 2021; 7(1): 111–8. doi: 10.1016/j.aninu.2020.10.004 33997338 PMC8110849

[32] Tan CQ, Li JY, Ji YC, Yang YY, Zhao XC, Chen MX, et al. Effects of dietary supplementation of different amounts of yeast extract on oxidative stress, milk components, and productive performance of sows. Anim Feed Sci Technol 2021; 274: 114648. doi: 10.1016/j.anifeedsci.2020.114648

[33] Ardiansyah, Inagawa Y, Koseki T, Agista AZ, Ikeda I, Goto T, et al. Adenosine and adenosine-5 ‘-monophosphate ingestion ameliorates abnormal glucose metabolism in mice fed a high-fat diet. BMC Complement Altern Med 2018; 18: 304. doi: 10.1186/s12906-018-2367-6. PubMed PMID: WOS:000450532100002. 30428888 PMC6236947

[34] Fukumori Y, Takeda H, Fujisawa T, Ushijima K, Onodera S, Shiomi N. Blood glucose and insulin concentrations are reduced in humans administered sucrose with inosine or adenosine. J Nutr 2000; 130(8): 1946–9. PubMed PMID: BIOSIS:PREV200000382331. 10917906 10.1093/jn/130.8.1946

[35] Cha SH, Wolfgang M, Tokutake Y, Chohnan S, Lane MD. Differential effects of central fructose and glucose on hypothalamic malonyl-CoA and food intake. Proc Natl Acad Sci U S A 2008; 105(44): 16871–5. doi: 10.1073/pnas.0809255105. PubMed PMID: WOS:000260913800016. 18971329 PMC2579345

[36] Pimentel GD, Ropelle ER, Rocha GZ, Carvalheira JBC. The role of neuronal AMPK as a mediator of nutritional regulation of food intake and energy homeostasis. Metabolism 2013; 62(2): 171–8. doi: 10.1016/j.metabol.2012.07.001. PubMed PMID: WOS:000314436300001. 22898253

[37] Salati LM, Gross CJ, Henderson LM, Savaiano DA. Absorption and metabolism of adenine, adenosine-5’-monophosphate, adenosine and hypoxanthine by the isolated vascularly perfused rat small intestine. J Nutr 1984; 114(4): 753–60. PubMed PMID: MEDLINE: 6716178. 6716178 10.1093/jn/114.4.753

[38] Farmer SR. Transcriptional control of adipocyte formation. Cell Metab 2006; 4(4): 263–73. doi: 10.1016/j.cmet.2006.07.001. PubMed PMID: WOS:000241182900004. 17011499 PMC1958996

[39] Rosen ED, Spiegelman BM. Adipocytes as regulators of energy balance and glucose homeostasis. Nature 2006; 444(7121): 847–53. doi: 10.1038/nature05483. PubMed PMID: WOS:000242805400041. 17167472 PMC3212857

[40] Gao J, Xiong R, Xiong D, Zhao W, Zhang S, Yin T, et al. The adenosine monophosphate (AMP) analog, 5-aminoimidazole-4-carboxamide ribonucleotide (AICAR) inhibits hepatosteatosis and liver tumorigenesis in a high-fat diet murine model treated with diethylnitrosamine (DEN). Med Sci Monit 2018; 24: 8533–43. doi: 10.12659/msm.910544. PubMed PMID: WOS:000451809200002. 30474622 PMC6278641

[41] Blondin DP, Labbe SM, Noll C, Kunach M, Phoenix S, Guerin B, et al. Selective impairment of glucose but not fatty acid or oxidative metabolism in brown adipose tissue of subjects with type 2 diabetes. Diabetes 2015; 64(7): 2388–97. doi: 10.2337/db14-1651. PubMed PMID: WOS:000356934000022. 25677914

[42] Fuller-Jackson JP, Henry BA. Adipose and skeletal muscle thermogenesis: studies from large animals. J Endocrinol 2018; 237(3): R99–115. doi: 10.1530/joe-18-0090. PubMed PMID: WOS:000438182500002. 29703782

[43] Townsend KL, An D, Lynes MD, Huang TL, Zhang HB, Goodyear LJ, et al. Increased mitochondrial activity in BMP7-treated brown adipocytes, due to increased CPT1- and CD36-mediated fatty acid uptake. Antioxid Redox Signal 2013; 19(3): 243–57. doi: 10.1089/ars.2012.4536. PubMed PMID: WOS:000321040300004. 22938691 PMC3691916

[44] Feldmann HM, Golozoubova V, Cannon B, Nedergaard J. UCP1 ablation induces obesity and abolishes diet-induced thermogenesis in mice exempt from thermal stress by living at thermoneutrality. Cell Metab 2009; 9(2): 203–9. doi: 10.1016/j.cmet.2008.12.014. PubMed PMID: WOS:000263269400011. 19187776

[45] Ji S, You Y, Kerner J, Hoppel CL, Schoeb TR, Chick WSH, et al. Homozygous carnitine palmitoyltransferase 1b (muscle isoform) deficiency is lethal in the mouse. Mol Genet Metab 2008; 93(3): 314–22. doi: 10.1016/j.ymgme.2007.10.006. PubMed PMID: WOS:000253603300122. 18023382 PMC2270477

[46] Barbera MJ, Schluter A, Pedraza N, Iglesias R, Villarroya F, Giralt M. Peroxisome proliferator-activated receptor alpha activates transcription of the brown fat uncoupling protein-1 gene: a link between regulation of the thermogenic and lipid oxidation pathways in the brown fat cell. J Biol Chem 2001; 276(2): 1486–93. doi: 10.1074/jbc.M006246200. PubMed PMID: BIOSIS:PREV200100226156. 11050084

[47] Singh AK, Aryal B, Chaube B, Rotllan N, Varela L, Horvath TL, et al. Brown adipose tissue derived ANGPTL4 controls glucose and lipid metabolism and regulates thermogenesis. Mol Metab 2018; 11: 59–69. doi: 10.1016/j.molmet.2018.03.011. PubMed PMID: WOS:000439548700005. 29627378 PMC6001401

[48] Bartelt A, Bruns OT, Reimer R, Hohenberg H, Ittrich H, Peldschus K, et al. Brown adipose tissue activity controls triglyceride clearance. Nat Med 2011; 17(2): 200-U93. doi: 10.1038/nm.2297. PubMed PMID: WOS:000286969900028. 21258337

[49] JohanssonA SM, Lindgren E, Yang J-N, Herling AW, Fredholm BB. Adenosine A(1) receptors regulate lipolysis and lipogenesis in mouse adipose tissue – interactions with insulin. Eur J Pharmacol 2008; 597(1–3): 92–101. doi: 10.1016/j.ejphar.2008.08.022. PubMed PMID: WOS:000260920300015. 18789919

[50] Dhalla AK, Santikul M, Smith M, Wong M-Y, Shryock JC, Belardinelli L. Antilipolytic activity of a novel partial A(1) adenosine receptor agonist devoid of cardiovascular effects: comparison with nicotinic acid. J Pharmacol Exp Ther 2007; 321(1): 327–33. doi: 10.1124/jpet.106.114421. PubMed PMID: WOS:000244989600036. 17204748

