# Supplementary material for: Dietary adenosine 5’-monophosphate supplementation increases food intake and remodels energy expenditure in mice
Source: Food Nutr Res. 2022 Jun 30;66:10.29219/fnr.v66.7680. doi: 10.29219/fnr.v66.7680 (PMC9250134; doi:10.29219/fnr.v66.7680)
Supplement: Dietary adenosine 5’-monophosphate supplementation increases food intake and remodels energy expenditure in mice [file FNR-66-7680-s001.docx]

**Supplementary Information**

**Supplementary Figures and Supplementary Table.**


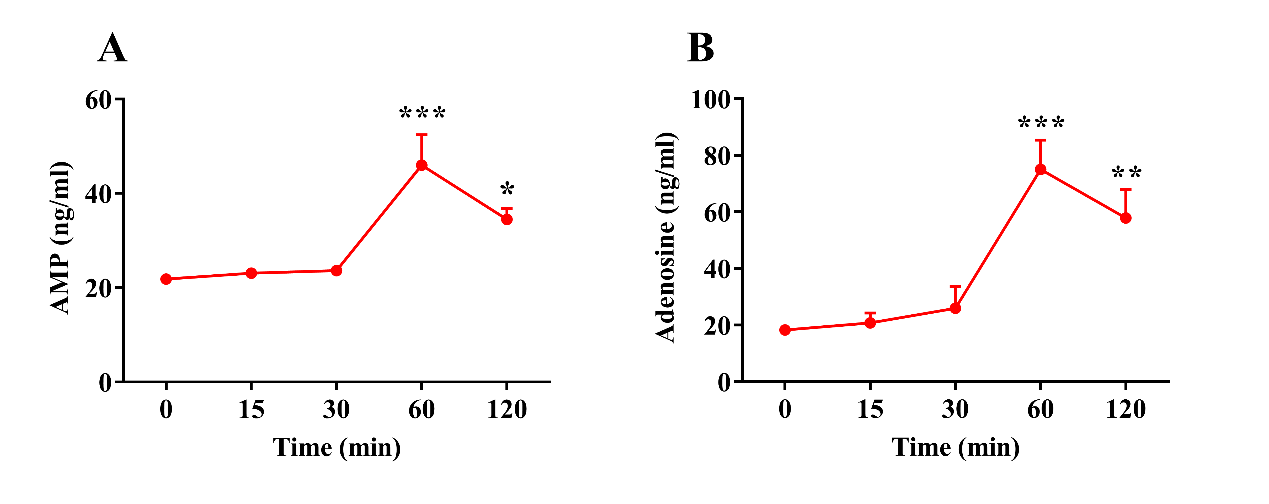


**Supplementary Figure S1.** Effect of AMP oral gavage on the content of serum AMP (A) and adenosine (B). Data are mean ± SEM (n = 5-7). Statistical analyses were performed using one-way ANOVA followed by Dunnett’s multiple comparisons test. * *P* < 0.05, ** *P* < 0.01, *** *P* < 0.001 *versus* the content at 0 min. AMP, adenosine 5’-monophosphate.

**Supplementary Table S1. Primer sets used for Real-Time PCR.**

| NCBI accession ID | Gene | Primer sequence （5’ to 3’） | Size(bp) |
| --- | --- | --- | --- |
| [XM_030254057.1](https://www.ncbi.nlm.nih.gov/entrez/viewer.fcgi?db=nucleotide&id=1720411884) | β-actin | F: GTCCCTCACCCTCCCAAAAG  R: GCTGCCTCAACACCTCAACCC | 266 |
| [XM_021170845.1](https://www.ncbi.nlm.nih.gov/entrez/viewer.fcgi?db=nucleotide&id=1195735408) | UCP1 | F: GCTTTGCCTCACTCAGGATTGG  R: CCAATGAACACTGCCACACCTC | 133 |
| [XM_006503779.4](https://www.ncbi.nlm.nih.gov/entrez/viewer.fcgi?db=nucleotide&id=1720412479) | PGC-1α | F: AGCCGTGACCACTGACAACGAG  R: GCTGCATGGTTCTGAGTGCTAAG | 168 |
| [XM_021184295.2](https://www.ncbi.nlm.nih.gov/entrez/viewer.fcgi?db=nucleotide&id=1679823686) | CPT-1β | F: ATGTATCGCCGCAAACTGGACC  R: CTCTGAGAGGTGCTGTAGCAAG | 147 |
| [XM_030248424.1](https://www.ncbi.nlm.nih.gov/entrez/viewer.fcgi?db=nucleotide&id=1720383712) | PPARα | FP: ATGCCAGTACTGCCGTTTTC  RP: GGCCTTGACCTTGTTCATGT | 220 |
| [XM_006505743.4](https://www.ncbi.nlm.nih.gov/entrez/viewer.fcgi?db=nucleotide&id=1720417315) | PPARγ | F: GTACTGTCGGTTTCAGAAGTGCC  R: ATCTCCGCCAACAGCTTCTCCT | 102 |
| [XM_006539175.4](https://www.ncbi.nlm.nih.gov/entrez/viewer.fcgi?db=nucleotide&id=1720409839) | PRDM16 | F: GGCGAGGAAGCTAGCCAAA  R: GGTCTCCTCCTCGGCACTCT | 97 |
| [XM_030255219.1](https://www.ncbi.nlm.nih.gov/entrez/viewer.fcgi?db=nucleotide&id=1720417278) | NRF1 | F: GGCAACAGTAGCCACATTGGCT  R: GTCTGGATGGTCATTTCACCGC | 141 |
| [XM_017313918.2](https://www.ncbi.nlm.nih.gov/entrez/viewer.fcgi?db=nucleotide&id=1720361181) | TFAM | F: CCTGAGGAAAAGCAGGCATA  R: ATGTCTCCGGATCGTTTCAC | 143 |
| [XM_021204930.2](https://www.ncbi.nlm.nih.gov/entrez/viewer.fcgi?db=nucleotide&id=1686472009) | SIRT1 | F: CCTGACTTCAGATCAAGAGA  R: TGTCTCCACGAACAGCTTCA | 349 |
| [XM_021179194.2](https://www.ncbi.nlm.nih.gov/entrez/viewer.fcgi?db=nucleotide&id=1679817909) | Dio2 | F: CCTCCTAGATGCCTACAAACAGG  R: CATTCGGCCCCATCAGCGGTC | 126 |
| [XM_021169248.2](https://www.ncbi.nlm.nih.gov/entrez/viewer.fcgi?db=nucleotide&id=1679856318) | LPL | F: GCCCAGCAACATTATCCAGT  R: GGTCAGACTTCCTGCTACGC | 168 |
| [NM_025802.3](https://www.ncbi.nlm.nih.gov/entrez/viewer.fcgi?db=nucleotide&id=254826781) | ATGL | F: AAAGGACCTGATGACCACC  R: GCAGCCACTCCAACAAGC | 125 |
| [NM_024406.3](https://www.ncbi.nlm.nih.gov/entrez/viewer.fcgi?db=nucleotide&id=1276740364) | FABP4 | F: TCACCTGGAAGACAGCTCCT  R: AATCCCCATTTACGCTGATG | 182 |
| [XM_021168520.2](https://www.ncbi.nlm.nih.gov/entrez/viewer.fcgi?db=nucleotide&id=1679854598) | C/EBPα | F: AGAAGTCGGTGGACAAGAACA  R: TTTGGCTTTATCTCGGCTCT | 89 |
| [XM_030245748.1](https://www.ncbi.nlm.nih.gov/entrez/viewer.fcgi?db=nucleotide&id=1720364972) | SREBP1 | F: CGACTACATCCGCTTCTTGCAG  R: CCTCCATAGACACATCTGTGCC | 143 |
| [XM_021212956.2](https://www.ncbi.nlm.nih.gov/entrez/viewer.fcgi?db=nucleotide&id=1686218660) | P2X1 | F: CTTTGGCTGGTGTCCTGTAGAG  R: CCTGTTGACCTTGAAGCGTGGA | 124 |
| [XM_030254197.1](https://www.ncbi.nlm.nih.gov/entrez/viewer.fcgi?db=nucleotide&id=1720412434) | P2X4 | F: GCTTTCAGGAGATGGCAGTGGA  R: TGTAGCCAGGAGACACGTTGTG | 153 |
| [NM_033321.4](https://www.ncbi.nlm.nih.gov/entrez/viewer.fcgi?db=nucleotide&id=1781908973) | P2X5 | F: AGAGGACAAGCCACTGGAGA  R: GTGATGGCTTCATGTTCACG | 152 |
| [XM_021184811.1](https://www.ncbi.nlm.nih.gov/entrez/viewer.fcgi?db=nucleotide&id=1195693378) | P2X6 | F: TGCTAACCAGGAACTGTCGGGT  R: AAGTCCCGTTCCTGGTAGCCTT | 115 |
| [XM_006529079.3](https://www.ncbi.nlm.nih.gov/entrez/viewer.fcgi?db=nucleotide&id=1720353437) | A1 | F: GATCGGTACCTCCGAGTCAAGA  R: CACTCAGGTTGTTCCAGCCAAAC | 142 |
| [XM_030244829.1](https://www.ncbi.nlm.nih.gov/entrez/viewer.fcgi?db=nucleotide&id=1720360109) | A_2_A | F: AGAGCAAGAGGCAGGTATCTC  R: CCCAAAGGCTTTCTCACGGA | 113 |

F: forward primer; R: reverse primer
